# Data of unhealthy food availability in hospitals

**DOI:** 10.1016/j.dib.2018.10.084

**Published:** 2018-11-03

**Authors:** Colin E. Champ, Nick A. Iarrobino, Christopher P. Haskins

**Affiliations:** a Department of Radiation Oncology, University of Pittsburgh Medical Center, Pittsburgh, PA, United States; b University of Pittsburgh School of Medicine, Pittsburgh, PA, United States; c Department of Internal Medicine, Henry Ford Hospital, Detroit, MI, United States; d Department of Radiation Oncology, University of Maryland, Baltimore, MD, United States

## Abstract

In our manuscript, we present the food choices available at vending machines in government-run Veterans Affairs Hospitals. The data in this article includes both a quantification of the beverages and packages foods available, along with a comparison of recommendations and sugar content to the government-issued USDA Dietary Guidelines 2015–2020. For further discussion on the results of this study, refer to the full manuscript “Lead by Poor Example: An Assessment of Snacks, Soda, and Junk Food Availability in Veterans Affairs Hospitals” (Champ et al., 2018) [1].

**Specifications table**

**Table:** 

Subject area	*Medicine*
More specific subject area	*Nutrition*
Type of data	*Tables and Figures*
How data was acquired	*Freedom of Information Act request through online submission*
Data format	*Raw*
Experimental factors	*The number and contents of vending machines were quantified at Veterans Affairs Hospitals, and nutritional content was calculated.*
Experimental features	*Data was compared with official government dietary guidelines, the USDA Dietary Guidelines 2015–2020, to assess whether the foods presented within their facilities correlated with these guidelines.*
Data source location	*All Veterans Affairs Hospital Locations, USA*
Data accessibility	*Data can be found within this article*

**Value of the data**

• These data reveal what foods are available in vending machines within our government-run Veterans Affairs Hospitals
• Findings illustrate the quantity of vending machines at all hospital locations
• These data expose the quantitative and qualitative aspects of required food and drink items offered to patients and guests within hospitals
• These data can be directly compared to government-provided dietary recommendations to assess consistency and areas that need addressed

## 1 Data

**Fig. 1:**
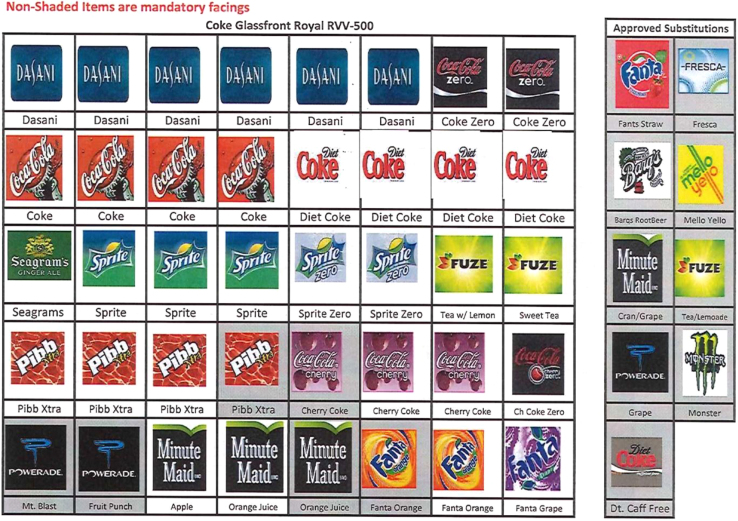
Example of required beverages in vending machines.

**Fig. 2:**
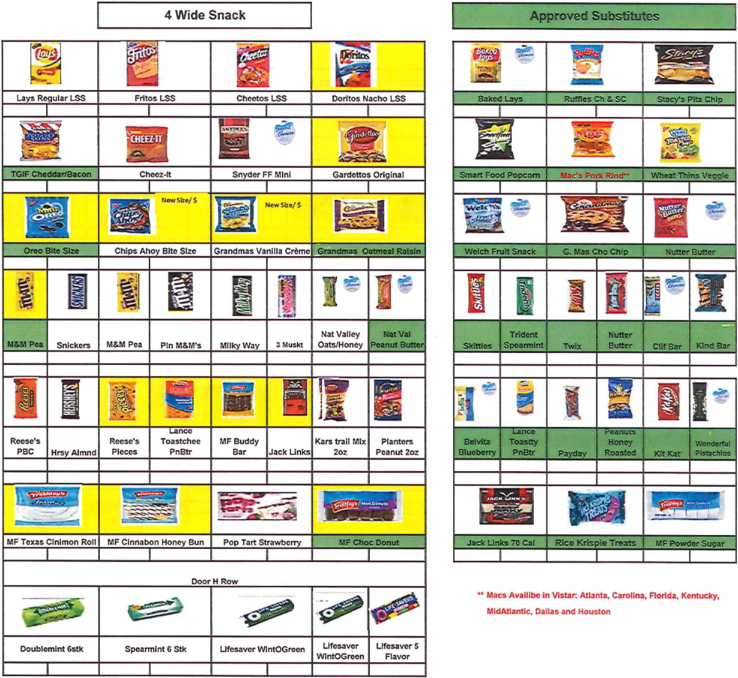
Example of required packaged foods in vending machines.

There are three tables describing the locations and contents of vending machines, and two figures revealing the mandated contents in each. Table 1 lists the Veteran Affairs facilities and number of vending machines on site. Table 2 describes the drink items supplied in vending machines and their sugar content. Table 3 describes the packaged food items available in vending machines. Fig. 1 is an example of required beverages in vending machines. Fig. 2 is an example of required packaged foods in vending machines.

**Table 1:** Veteran Affairs facilities and number of vending machines on site.

**Location**	**#**	**Location**	**#**	**Location**	**#**	**Location**	**#**
Albany, NY	16	Dayton, OH	27	Long Beach CA	26	Reno, NV	8
Albuquerque, NM	20	Decatur, GA	22	Los Angeles CA	34	Richmond, VA	25
Alexandria, LA	18	Denver, CO	11	Los Angeles, CA	15	Roseburg, OR	11
Altoona, PA	7	Des Moines, IA	10	Los Angeles, CA OPC	3	Sacramento, CA	6
Amarillo, TX	15	Detroit, MI	18	Louisville, KY	16	Saginaw, MI	8
Ann Arbor, MI	30	Dublin, GA	15	Lyons, NJ	19	Salem, VA	17
Asheville, NC	25	Durham, NC	19	Madison, WI	19	Salisbury, NC	21
Aspinwall, PA	11	East Orange, NJ	23	Manchester, NH	8	Salt Lake City, UT	22
Augusta, GA (DT)	15	El Paso, TX	12	Marion, IL	17	San Antonio, TX	23
Augusta, GA U	14	Erie, PA	6	Marion, IN	8	San Diego, CA	19
Austin, TX	1	Fargo, ND	15	Martinez, CA	8	San Francisco, CA	15
Baltimore, MD	5	Fayetteville, AR	7	Martinsbrug, WV	23	San Juan, PR	38
Batavia, NY	4	Fayetteville, NC	11	Melbourne, FL	2	Seattle, WA	10
Bath, NY	13	Fort Meade, SD	9	Memphis, TN	28	Sepulveda, CA	7
Battle Creek, MI	20	Fort Wayne, IN	8	Menlo Park CA	10	Sheridan, WY	19
Bay Pines, FL	39	Fresno, CA	11	Miami, FL	28	Shreveport, LA	19
Beckley, WV	12	Ft Harrison, MT	5	Middleton, WI	2	Sioux Falls, SD	6
Bedford, MA	31	Ft Worth, TX	4	Milwaukee, WI	31	Spokane, WA	4
Big Spring, TX	8	Ft McPherson, GA	4	Minneapolis, MN	39	St Louis, MO JB	17
Biloxi, MS	27	Gainesville, FL	26	Montgomery, AL	11	St Louis, MO JC	16
Birmingham, AL	18	Grand Island, NE	5	Montrose, NY	16	St. Albans, NY	9
Boise, ID	11	Grand Junction, CO	4	Mountain Home, TN	28	Syracuse, NY	14
Bonham, TX	13	Hampton, VA	18	Murfreesboro, TN	21	Tacoma, WA	13
Boston, MA	18	Hines, IL	56	Muskogee, OK	14	Tampa FL	20
Brockton, MA	13	Hot Springs, SD	8	N Little Rock, AR	22	Temple, TX	33
Bronx, NY	25	Houston, TX	43	Nashville, TN	16	Togus, ME	13
Brooklyn, NY	6	Huntington, WV	20	New York, NY	14	Tomah, WI	27
Buffalo, NY	17	Indianapolis, IN	16	Newington, CT	5	Topeka, KS	18
Canadaigua, NY	14	Iowa City, IA	18	North Chicago, IL	29	Tucson, AZ	25
Castle Point, NY	6	Iron Mountain, MI	9	Northampton, MA	5	Tuscaloosa, AL	20
Charleston, SC	13	Jackson, MS	20	Northport, NY	21	Tuskegee, AL	11
Cheyenne, WY	7	Kansas City, MO	17	Oklamhoma City, OK	20	Vancouver, WA	14
Chicago, IL (WS)	25	Kerrville, TX	5	Omaha, NE	17	Waco, TX	21
Chillicothe, OH	27	Lake City, FL	17	Orlando, FL	8	Walla Walla, WA	8
Cincinatti, OH	17	Lake Nona, FL	9	Palo Alto, CA	35	Washington, DC	18
Clarksburg, WV	6	Las Vegas, NV	39	Perry Point, MD	17	West Haven, CT	18
Cleveland, OH	33	Leavenworth, KS	29	Philadelphia, PA	13	West Palm Beach, FL	16
Coatesville, PA	30	Lebanon, PA	18	Phoenix, AZ	24	West Roxbury, MA	12
Columbia, MO	15	Leestown, KY	10	Pittsburgh, PA	24	White City, OR	16
Columbia, SC	19	Lexington, KY	13	Poplar Bluff, MO	8	White River JCT, VT	7
Columbia, OH	7	Little Rock, AR	10	Portland, OR	14	Wichita, KS	19
Dallas, TX	57	Loch Raven MD	6	Prescott, AZ	12	Wilkes-Barre, PA	9
Danville, IL	20	Loma Linda, CA	20	Providence, RI	10	Wilmington, DE	11

# = number of vending machines at facility.

**Table 2:** Drink items supplied in vending machines and their sugar content.

**Soda machine**	**Mandatory item**	**Sugar (g) in 12 oz. portion**	**Sugar (g) in 20 oz. portion**	**Substitution item**	**Sugar (g) in 12 oz. portion**	**Sugar (g) in 20 oz. portion**
1. Coke Glassfront Royal RVV-500 2. 2. Coke Glassfront Model 5800, Vue 40^a^	Dasani Water	0	0	Fanta Strawberry	44	74
	Coca-Cola	39	65	Fresca	0	0
	Coca Cola Zero	0	0	Barg׳s Root Beer	45	75
	Diet Coke	0	0	Mello Yellow	47	78
	Sprite Zero	0	0	Minute Maid Cranberry Grape Juice	45	N/A
	Fuze Sweet Tea	44	N/A	Poweraid	N/A	34
	Coca-Cola Cherry	42	70	Monster Energy Drink	55^b^	
	Seagrams Ginger Ale	33	55	Diet Caffeine Free Coke	0	0
	Sprite	38	57	Fuze Sweet Tea Lemonade	19	31
	Pibb Xtra	39	65			
	Minute Maid Apple Juice	48	N/A			
	Minute Maid Orange Juice	40	N/A			
	Fanta Orange	44	74			
	Fanta Grape	44	74			
						
1. Pepsi Glassfront Stand Alone Model 5800, Vue 40	Aquafina	0	0	Sierra Mist	37	62
	Pepsi Max	0	0	Orange Crush	43	71
	Pepsi	41	69	Grape Crush	43	71
	Diet Pepsi	0	0	Mountain Dew Red	46	77
	Mountain Dew	46	77	Diet Caffeine-Free Pepsi	0	0
	Diet Mountain Dew	0	0	Orange Gatorade	21	34
	Mug Root Beer	43	71	Lemon/Lime Gatorade	22	34
	Brisk Iced Tea	17	29	Cool Blue Gatorade	21	34
	Lipton Green Tea	N/A	32	Starbucks Mocha Frap	31^c^	N/A
	Diet Green Tea	0	0	Starbucks Vanilla Frap	31^c^	N/A
	Canada Dry/Scwepps Ginger Ale	32	54	Ocean Spray Crangrape	56^a^	N/A
	Gatorade Fruit Punch	21	34	Ocean Spray Cranberry~	37^d^	N/A
	Ocean Spray Orange~	44^a^	N/A	Ocean Spray Grapefruit~	42	N/A
	Dr. Pepper	41	66	AMP Energy Drink	N/A	58^b^
	Diet Dr. Pepper	0	0	AMP Sugar Free	N/A	0
				Diet Cherry Pepsi	0	0

N/A = not available, oz = fluid ounce.

a Option of adding Dr. Pepper (12 oz 41 g and 20 oz 66 g sugar) and Diet Dr. Pepper.

b 16 oz, ~100% fruit juice.

c 9.5 oz.

d 10 oz.

**Table 3:** Food items available in vending machines.

**Mandatory Items**	**Substituted Items**
**Candy:**	**Candy:**
M&Ms	Skittles
Peanut M&Ms	Twix Candy Bar
Snickers Candy Bar	Payday Candy Bar
Milky Way Candy Bar	Kit Kat Candy Bar
3 Musketeer Candy Bar	Twizzlers
Reese׳s Peanut Butter Cup	Almond Joy Candy Bar
Reese׳s Pieces Candy	**Potato Chips/Puffed Corn Snack**
Hershey Almond Bar	Baked Lays Potato Chips
MF Buddy Bar	Ruffles Cheese & Sour Cream Potato Chips
Welch׳s Fruit Snack	Stacey׳s Pita Chips
Lifesaver Wintogreen	Lays Barbecue Potato Chips
Lifesaver 5	Sweet Potato Kettle Chips
**Pastry/Frosted Baked Goods**	**Nuts/Trail Mix**
MF Texas Cinnamon Roll	Kind Bar
MF Cinnabon Honey Bun	Honey Roasted Peanuts
Strawberry Pop Tart	Wonderful Pistachios
Rice Krispie Treats	**Cookies**
MF Chocolate Donut	Grandmas Chocolate Chip Cookies
**Cookies**	Nutter Butter Cookies
Oreo Bite Size	**Crackers/Pretzels**
Chips Ahoy Bite Size	Wheat Thins
Grandmas Vanilla Crème Cookies	Wheat Thins Veggie
Grandmas Oatmeal Raisin Cookies	**Pastry/Frosted Baked Goods**
**Potato Chips/Puffed Corn Snack**	Belvita Blueberry
Lays Regular Potato Chips	MF Powdered Sugar Donuts
Fritos	**Gum**
Cheetos	Trident Spearmint
Doritos Nacho	Trident Tropical Twist
**Crackers/Pretzels**	**Popcorn**
TGIF Cheddar/Bacon	Smart Popcorn
Cheez-It	Act II Popcorn
Snyder Mini Pretzels	**Pork Rinds**
Gardettos Original	Mac׳s Pork Rinds
Lance Toast Chee Peanutbutter Crackers	**Granola Bar**
**Granola Bar**	Clif Bar
Nature Valley Oats and Honey	**Jerky**
Nature Valley Peanut Butter	Jack Links Tender Cut Jerky
	
**Nuts/Trail Mix**	
Kars Trail Mix	
Planters Peanuts

## 2 Experimental design, materials and methods

Veterans Affairs Hospitals were assessed via an official request after process verification for the Freedom of Information Act from the Veterans Administration. The request was for the location and number of VA hospitals containing vending machines, the number of vending machines within each hospital, and the contents of these vending machines. The contents were analyzed from the FOIA reports, and the nutritional contents of each beverage were assessed via the United States Department of Agriculture (USDA) Food Composition Databases, pepsicobeveragefacts.com, coca-colaproductfacts.com, and myfitnesspal.com if the former sources did not contain nutritional information. These results were compared with official government dietary guidelines, the USDA Dietary Guidelines 2015–2020.
