# Supplementary material for: Data of unhealthy food availability in hospitals
Source: Data Brief. 2018 Nov 3;21:1738–44. doi: 10.1016/j.dib.2018.10.084 (PMC6249542; doi:10.1016/j.dib.2018.10.084)
Supplement: Supplementary file 1 — Supplementary material [file mmc1.docx]

The authors report no financial conflicts of interest.
